# Data quality considerations for evaluating COVID-19 treatments using real world data: learnings from the National COVID Cohort Collaborative (N3C)

**DOI:** 10.1186/s12874-023-01839-2

**Published:** 2023-02-17

**Authors:** Hythem Sidky, Jessica C. Young, Andrew T. Girvin, Eileen Lee, Yu Raymond Shao, Nathan Hotaling, Sam Michael, Kenneth J. Wilkins, Soko Setoguchi, Michele Jonsson Funk, The N3C Consortium, G. Caleb Alexander, Benjamin Bates, Christopher G. Chute, Jayme L. Dahlin, Ken Gersing, Melissa A. Haendel, Hemalkumar B. Mehta, Emily R. Pfaff, David Sahner

**Affiliations:** 1 National Center for Advancing Translational Sciences, National Institutes of Health, Bethesda, MD USA; 2 Axle Research and Technologies, Rockville, MD USA; 3 Cecil G. Sheps Center for Health Services Research, The University of North Carolina at Chapel Hill, Chapel Hill, NC USA; 4 Palantir Technologies, Denver, CO USA; 5 Rutgers Robert Wood Johnson Medical School, Rutgers University, New Brunswick, NJ USA; 6 Duke University Medical Center, Durham, NC USA; 7 National Institute of Diabetes & Digestive & Kidney Diseases, Office of the Director, National Institutes of Health, Bethesda, MD USA; 8 F. Edward Hébert School of Medicine, Department of Preventive Medicine & Biostatistics, Uniformed Services University of the Health Sciences, Bethesda, MD USA; 9 Department of Epidemiology, Gillings School of Global Public Health, The University of North Carolina at Chapel Hill, Chapel Hill, NC USA

**Keywords:** COVID-10, SARS-CoV-2, EHR data, Pharmacoepidemiology, Data quality, Real world data

## Abstract

**Background:**

Multi-institution electronic health records (EHR) are a rich source of real world data (RWD) for generating real world evidence (RWE) regarding the utilization, benefits and harms of medical interventions. They provide access to clinical data from large pooled patient populations in addition to laboratory measurements unavailable in insurance claims-based data. However, secondary use of these data for research requires specialized knowledge and careful evaluation of data quality and completeness. We discuss data quality assessments undertaken during the conduct of prep-to-research, focusing on the investigation of treatment safety and effectiveness.

**Methods:**

Using the National COVID Cohort Collaborative (N3C) enclave, we defined a patient population using criteria typical in non-interventional inpatient drug effectiveness studies. We present the challenges encountered when constructing this dataset, beginning with an examination of data quality across data partners. We then discuss the methods and best practices used to operationalize several important study elements: exposure to treatment, baseline health comorbidities, and key outcomes of interest.

**Results:**

We share our experiences and lessons learned when working with heterogeneous EHR data from over 65 healthcare institutions and 4 common data models. We discuss six key areas of data variability and quality. (1) The specific EHR data elements captured from a site can vary depending on source data model and practice. (2) Data missingness remains a significant issue. (3) Drug exposures can be recorded at different levels and may not contain route of administration or dosage information. (4) Reconstruction of continuous drug exposure intervals may not always be possible. (5) EHR discontinuity is a major concern for capturing history of prior treatment and comorbidities. Lastly, (6) access to EHR data alone limits the potential outcomes which can be used in studies.

**Conclusions:**

The creation of large scale centralized multi-site EHR databases such as N3C enables a wide range of research aimed at better understanding treatments and health impacts of many conditions including COVID-19. As with all observational research, it is important that research teams engage with appropriate domain experts to understand the data in order to define research questions that are both clinically important and feasible to address using these real world data.

**Supplementary Information:**

The online version contains supplementary material available at 10.1186/s12874-023-01839-2.

## Background

Given the unprecedented global spread and impact of COVID-19, researchers are urgently conducting research to understand the safety and effectiveness of treatment options [1, 2] and to understand short and long term sequelae of SARS-CoV-2 infection [3]. Due to time exigencies and financial constraints, ethical considerations, and the suitability of certain lines of inquiry, randomized clinical trials are not always feasible or necessary, and research using existing secondary data can offer valuable hypothesis-generating insights or reliable evidence as medical professionals respond to the pandemic [4]. In addition, inclusion of data from a wide array of institutions increases sample size enabling researchers to study less common conditions or treatments, and can enhance generalizability of results. Electronic health records (EHR) are generated at the time of healthcare delivery as a component of clinical care. Structured data in EHR typically include detailed information on clinical encounters including any procedures, diagnoses, ordered and administered medications, demographic data, vitals, and lab orders and results. Because the United States does not have a universal healthcare system, EHR data are maintained by individual health systems, each with different standards and protocols for data collection and storage, leading to a high degree of variability in the availability and quality of data across systems. Even when the same EHR platform is used by two health systems, differences in implementation are common. This lack of centralized or standardized reporting creates difficulties in using EHR at a large scale to conduct nationally representative research.

To enable COVID-19 research driven by data acquired across the United States, the National Center for Advancing Translational Sciences (NCATS) supported the creation of the National COVID Cohort Collaborative (N3C), a centralized repository of EHR-sourced data currently including over 9 million patients from 69 sites representing 49 out of 50 states that can be leveraged to study potential treatments and evaluate standards of care and best practices for COVID-19 in a real-world setting [5]. Compared to census data, N3C data have been shown to be more racially diverse, though biased towards urban as opposed to rural areas [6]. N3C aggregates and harmonizes EHR data across clinical organizations in the United States and supports data from both harmonized and unharmonized common data models (CDMs) including ACT, OMOP, PCORnet, and TriNetX, with OMOP version 5.3.1 being the target data model into which others are converted. Both automated and manual data ingestion and harmonization protocols are in place which ensure source CDM conformance to specific requirements and fitness for use [7].

The establishment of N3C coincides with a growing interest in the use of non-trials-based real-world data (RWD) to inform public health policy, formulate testable hypotheses for designing randomized clinical trials, and assist in clinical decision making. Concomitantly, concerns have also been raised over published findings using RWD that can, at times, seem contradictory [8, 9]. Model and data harmonization efforts [7] in centralized EHR repositories are the first step towards answering many research questions. However, even harmonized records may require further cleaning and processing depending on a provider’s data capture practices and source data model. Furthermore, high-quality RWE study design requires high-quality data, which involves a close examination of all data streams and deep understanding of their limitations and the sources and mechanisms behind data quality issues, such as missingness [10, 11]. Only then can these data be used to develop studies that support public health, generate viable hypotheses, and aid in clinical decision making. In light of this, our objectives were to highlight several important areas to be examined to ensure high data quality and to present potential solutions and risk-mitigation strategies based on our experience.

## Methods

The N3C data enclave systematically aggregates EHR data from partnering health systems, known as data partners, for patients who have tested positive for COVID-19 or have equivalent diagnosis codes according to the N3C phenotype. Negative controls with a non-positive SARS-CoV-2 lab result are also included at a 1:2 ratio (cases:controls). The specifics of the N3C phenotype are detailed on the N3C Github [12]. The final pooled data set includes information on hospital admissions, procedures, diagnoses, medications, lab test results, demographics, and basic vitals. This research was possible because of the patients whose information is included within the data and the organizations (https://ncats.nih.gov/n3c/resources/data-contribution/data-transfer-agreement-signatories) and scientists who have contributed to the on-going development of this community resource. The N3C data transfer to NCATS is performed under a Johns Hopkins University Reliance Protocol #IRB00249128 or individual site agreements with the NIH. The N3C Data Enclave is managed under the authority of the NIH; information can be found at https://ncats.nih.goc/n3c/resources. The content is solely the responsibility of the authors and does not necessarily represent the official views of the National Institutes of Health or the N3C program. Use of N3C data for this study does not involve human subjects (45 CFR 46.102) as determined by the NIH Office of IRB Operations.

Our focus was to conduct an in-depth data quality investigation to inform best practices for using these data for public health research specifically focusing on in-hospital drug effectiveness studies. Medications received during an inpatient stay cannot be identified using insurance claims data due to the bundling of facility charges in which a flat fee is charged for nursing, medications, supplies, etc. during each day of hospitalization. Thus, EHR data are potentially valuable for evaluations of drug utilization, safety and effectiveness in the inpatient setting [13, 14]. We identify considerations relevant to a hypothetical study evaluating the efficacy of remdesivir treatment in hospitalized patients with COVID-19. We discuss how to appropriately define concepts of interest, highlight data quality considerations, and offer suggestions for researchers using centralized multi-institution EHR-sourced data repositories.

### Study population

As a base cohort for our motivating example, we identified adult patients (≥ 18 years) who were hospitalized with COVID-19 between March 1, 2020 and September 1, 2021. The index date (initial observation period) was the first of either laboratory confirmed SARS-CoV-2 or the presence of at least one of several “strong positive” COVID-19 related diagnosis, as defined by the N3C version 3.3 phenotype [12]. Visits were defined according to the macrovisit aggregation algorithm available in the N3C enclave which combines individual OMOP visit records that appear to be part of the same care experience [15]. This is crucial as clinical encounter data is highly heterogeneous at both the CDM and institutional level. We included only the first hospitalization visit for patients meeting the inclusion requirements. All overlapping inpatient and emergency department visits for a single patient were merged to reconstruct complete hospital stays. We excluded patients with missing age or sex information and excluded visits shorter than 2 days. We also excluded patients who had positive COVID-19 test results predating 1/1/2020, as earlier positive results are implausible. Systematic missingness and other quality concerns in patient data outlined below further excluded all but 12 data partners for our in-hospital treatment effectiveness study. Since data suitability can vary considerably depending on the research question of interest, we discuss the specific criteria for these inclusion in our analysis in the results below.

### Covariate definition

A key component of observational studies using EHR data is the operationalization of definitions of baseline health status and severity of illness. Characterizing this allows us to 1) compare different treatment options while accounting for patient characteristics, and 2) examine if the treatment effects vary across different types of patient populations. We use the term “covariate” to define a variable that describes a patient and any relevant concepts of interest (age, sex, presence of health comorbidities). We focus on “baseline” covariates, meaning we collect information on relevant characteristics using data from before the exposure of interest is measured [16]. Previous investigation has already provided evidence that these baseline characteristics, many of which are only available in EHR data, are highly predictive of overall disease course severity [17].

For this illustrative example, our list of covariates includes concepts that may be related to receipt of treatment for COVID-19 or risk of COVID-19 related outcomes, including patient demographics (age, sex, race, ethnicity), smoking status, patient body mass index (BMI), chronic comorbid conditions included in the Charlson comorbidity index (hypertension, diabetes, coronary artery disease, congestive heart failure, chronic obstructive pulmonary disease, cerebrovascular disease, chronic kidney disease, cardiac arrhythmia, malignancy), and prior medication use (angiotensin-converting enzyme or ACE-inhibitors, angiotensin receptor blockers or ARBs, statins) [18]. In addition to characterizing chronic health conditions, the N3C data include lab measurements offering more proximal indicators related to illness severity or prognosis. We collect data on creatinine, bilirubin, partial arterial oxygen pressure (PaO2), fraction of inspired oxygen (FiO2), body temperature, white blood count (WBC), ferritin, C-reactive protein (CRP), interleukin-6 (IL-6), oxygen saturation (SpO2), and respiration rate within 2 days of the date of admission.

### Data quality assessment

In the following sections, we describe our data quality findings. We first assess the proportion of missing data across covariates of interest by institution. A visual examination of the distribution of non-missing values is also carried out. Observations exceeding two standard deviations from the global mean are identified. We then explore drug exposures and duration of treatment, focusing on evidence of treatment with remdesivir or dexamethasone initiated within the first 2 days of admission for COVID-19. Within OMOP, drug exposures are individual records corresponding to when a drug was ordered or administered to a patient. The specifics of how these data are recorded depend on the source data model and provider practice. We discuss the nuances of these data and how they relate to operationalizing definitions of exposure to medications during hospitalization. We then assess EHR continuity and its importance in capturing longitudinal care and ensuring adequate history for baseline health status of the patients included in our analyses. Subsequently, we examine multiple clinical outcomes of interest relating to COVID-19 hospitalizations including mortality, invasive mechanical ventilation, acute inpatient events, and composite outcomes. We discuss considerations in defining these events, and implications they may have on research.

## Results

The following sections identify the main types of quality issues, the goals in addressing them, specific challenges encountered, and the approaches that were ultimately adopted. This is followed by suggestions for researchers who seek to effectively leverage EHR data aggregated from numerous healthcare systems.

### Data missingness and reconciliation

#### Goal

Use the N3C database to define a cohort of interest and all relevant concepts (exposures, outcomes, confounders of interest) to evaluate the effectiveness of COVID-19 treatments.

#### Challenge encountered

Multi-site EHRs, even those in which the CDM has been harmonized, can display a large degree of source-specific variability in data availability. In particular, some data partners may be limited in what they can contribute due to restrictions of their source data model, differences in their extract/transform/load (ETL) processes, use of multiple non-integrated EHR platforms, or other non-technical reasons. Even in data from high-completeness sources, missingness patterns can be particularly challenging to deal with. Therefore, identifying data completeness of the prognostic factors most relevant to the study is an important first step.

#### Approach

**Fig. 1:**
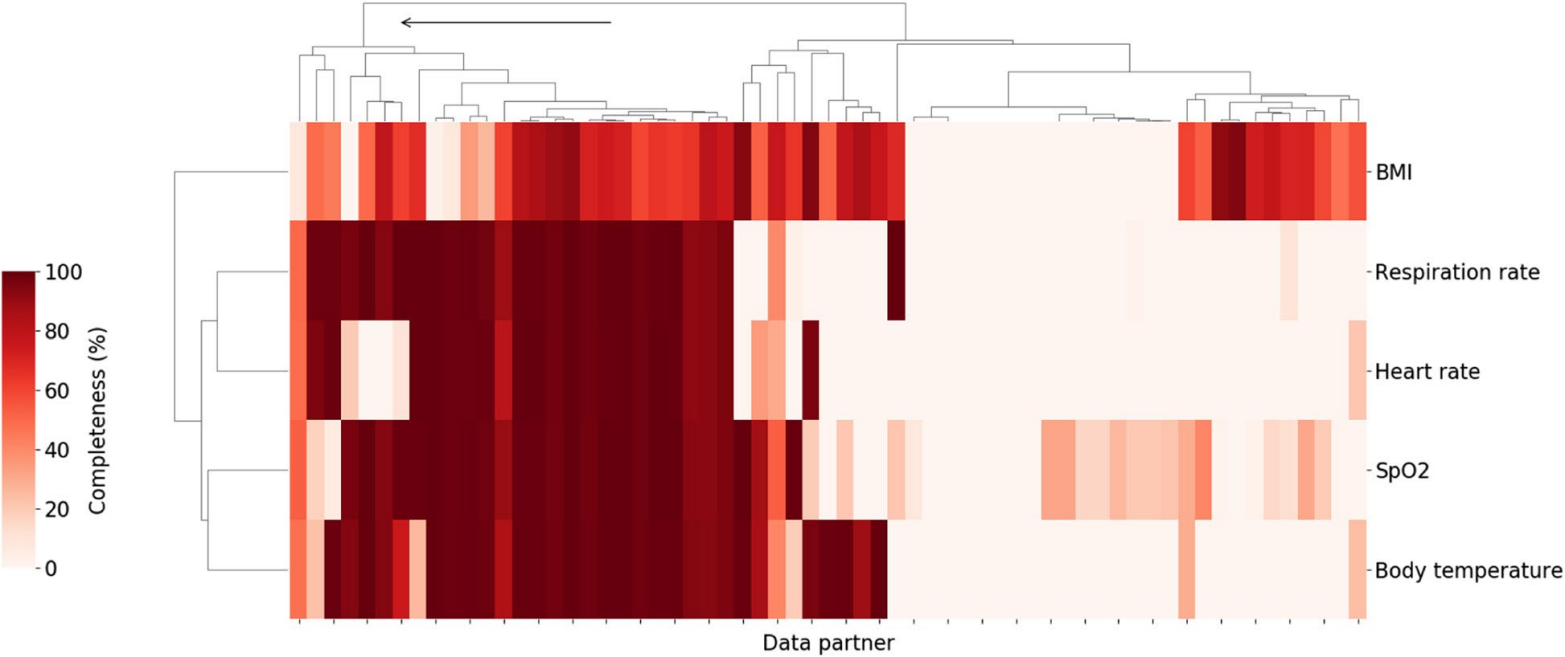
Percent of hospitalized COVID patients with key vitals at each of the N3C data partners. Darker colors indicate a higher percentage of patients with at least one measurement for the relevant vital sign during the course of their hospitalization. Arrow indicates branch of the cluster corresponding to retained data partners (26 total). Branches of the dendrogram which are closer together represent data partners which are more similar

Vital signs and laboratory measurements which have previously been associated with higher clinical severity within a COVID-19 related hospital encounter [17] were chosen for data completeness analysis. Starting with vitals, data partners with high levels of data completeness for oxygen saturation measurements, respiratory rate, body temperature, heart rate, and BMI were identified. Figure 1 shows the result of hierarchical clustering of data partners based on the percentage of patients hospitalized with COVID-19 who had at least one value for those variables. Individual data partners were found to vary significantly in the extent to which they record those measurements in the study population. For example, the proportion of patients with at least one recorded value for body temperature ranged from 0 to 100% with a median of 41.4% across 67 data partners. In some cases, this may be attributed to limitations inherent to the source data model used by the data partner. In other cases, data partners with data models that do support vitals are still missing significant portions of these data. Regardless of the cause, data partners with over 70% of missing data across these key measurements were excluded. This corresponded to retaining only those data partners belonging to the first top level cluster as shown in Fig. 1.

In addition to dropping sites with significant missing data, available metadata indicated that some data partners shifted dates prior to submission to the enclave despite being part of the HIPAA limited (LDS) data set which is expected to include non-shifted event dates. Given the dynamic nature of the pandemic, with evolving viral variants and changing standards of care, both of which may affect outcomes, only partners that did not shift their dates beyond a week were included in this study.

As a second step, the degree of capture of key variable values and the elements of critical concept sets was evaluated. A concept set is defined as the presence of one or more of a collection of diagnoses or observations, each of which define the same unified clinical concept. Since one of the unique advantages of EHR data is access to laboratory data, the discussion below is focused on clinical laboratory data, though much of it applies to demographic data and other covariates as well. Further attention was given to data and variables that were reasonably assumed to be missing not at random (MNAR) or missing at random (MAR); most ad hoc approaches for handling data using only “complete case” participants is tantamount to the very restrictive (and typically untenable) assumption that data are missing completely at random (MCAR). Therefore, it is imperative that analysts of EHR-based data make use of all individuals for a given data partner (including those with some incomplete data) and implement appropriate methods (e.g. multiple imputation, weighting) to address incomplete data capture within some individuals to better appeal to less restrictive MAR assumptions [19].

**Fig. 2:**
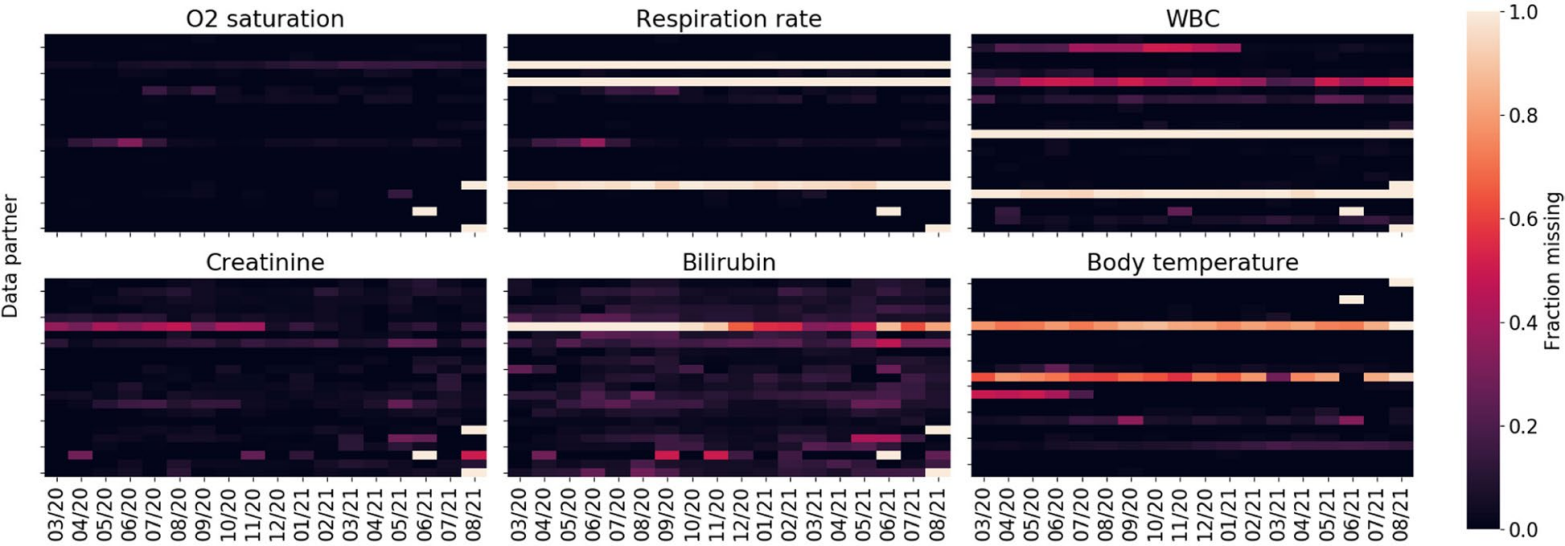
Fraction of missing data for key labs and measurements as a function of pandemic month and data partner

Figure 2 shows missingness of key labs and measurements among the population of hospitalized COVID-19 patients as a function of data partner and time. Temporal missingness patterns were evident, as was reporting heterogeneity among data partners. Some data partners did not appear to report certain key concepts at all. Due to the importance of these variables for establishing disease severity, data partners with (a) a proportion of missing data exceeding two standard deviations of the global proportion or (b) temporal variance exceeding two standard deviations of the global temporal variance were eliminated.

For the remaining data partners, Table 1 shows measurements for additional parameters of interest among the population of hospitalized COVID-19 patients, on a per-patient basis. Notably, substantial amounts of missingness across several measures that may be predictive of COVID-19 severity were seen. Interleukin-6 (IL-6) and oxygenation index (PaO2/FiO2) were missing in over 97% of hospitalizations and C-reactive protein and ferritin over 98% of the time; despite all four being identified as risk factors for COVID-19 related mortality [20]. This is not unsurprising as they are not collected routinely in the context of standard of care, and in fact are more likely to be collected or recorded for individuals in critical condition. However, data were also frequently missing for several parameters that would be expected to be available for patients hospitalized with COVID-19. These data underscore the difference between EHR data and data sets populated with results systematically collected in the context of prospective clinical trials and epidemiologic cohorts (study contexts with more clearly delineated best practice for accommodating missing values; for example, see the National Research Council panel report on clinical trials, https://pubmed.ncbi.nlm.nih.gov/24983040/ or the PCORI Methodology Committee standards).

**Table 1:** Characteristics of patients with a COVID-19 related hospitalization (*N* = 43,462). Each record represents a unique patient. Missingness is defined as the lack of any record of the variable of interest during the entire duration of the visit for that patient

Variable	Non-Missing	Missing (%)	Median value (IQR)
**BMI**	33,120	10,342 (23.8)	29 (25, 35)
**Creatinine**	18,610	24,852 (57.2)	0.94 (0.73, 1.40)
**Bilirubin**	8411	35,051 (80.6)	0.50 (0.37, 0.70)
**PaO2**	465	42,997 (98.9)	79 (71, 90)
**FiO2**	1047	42,415 (97.6)	0.69 (0.52, 0.83)
**Body temperature**	38,065	5397 (12.4)	36.8 (36.7, 37.0)
**WBC**	15,021	28,441 (65.4)	7.8 (5.6, 10.7)
**Ferritin**	781	42,681 (98.2)	555 (270, 1116)
**CRP**	1411	42,051 (96.8)	52 (23, 98)
**IL-6**	< 20	> 43,000 (100)	34.5 (32.6, 34.9)
**O2 saturation**	39,030	4432 (10.2)	95.0 (93.8, 96.5)
**Respiration rate**	39,041	4421 (10.2)	19.1 (18.0, 21.0)

Following this close examination of data missingness by data partner, 15 out of 67 sites (43,462 out of 307,193 patients) remained in our analysis. While retaining only 22% of data partners significantly reduces the sample size, the strict criteria ensured a more internally valid population, avoiding potential biases related to missing values’ impact on downstream analyses.

#### Takeaway / suggestions for researchers

When working with a large heterogeneous dataset that integrates data from many individual health systems, it is prudent to consider which hospital systems or data partners are appropriate for the analysis at hand, with their respective extent of data missingness as a key consideration. For each specific research question, it will be important to think critically about what will be important to measure, to evaluate how these variables and concepts are reported across data partners, and potentially exclude partners that do not meet these question-specific criteria.

### Drug exposure

### Drug code sets

#### Goal

Evaluate the use of therapeutic medications administered to hospitalized COVID-19 patients.

#### Challenge encountered

A significant amount of variability was found in drug exposure data due to flexibility in the data model specification. Depending on how the drug was coded, dosage information may or may not be available. If an active ingredient was coded at the ingredient level, dose and route of administration information were frequently missing with strength entirely missing. Continuing with the working example of remdesivir, Table 2 shows the distribution of drug concept names for the descendants of the RxNorm ingredient class code remdesivir. The vast majority (88.5%) are coded at the ingredient level with no available route of administration nor dosage available. This is not particularly problematic for the route of administration since remdesivir is only administered intravenously and has a well-defined dosage, though duration of therapy might vary in clinical practice.

**Table 2:** Distribution of drug concept names for Remdesivir in patient population

Drug concept name	N (%)
remdesivir	20,762 (88.5%)
remdesivir 100 MG Injection	2471 (10.5%)
remdesivir Injection	187 (0.80%)
20 ML remdesivir 5 MG/ML Injection	30 (0.13%)

These challenges, however, were more of an issue when looking at other drug exposures, such as dexamethasone, which can be administered in a variety of dosages, routes, and strengths (Table 3).

**Table 3:** Distribution of drug concept names for dexamethasone in patient population

Drug concept name	N (%)
dexamethasone	7442 (40.9%)
dexamethasone 2 MG Oral Tablet	6342 (34.8%)
dexamethasone 0.5 MG Oral Tablet [Decadron]	2179 (12.0%)
dexamethasone 0.5 MG Oral Tablet	1811 (9.94%)
dexamethasone 1 MG/ML Oral Solution [Dexamethasone Intensol]	166 (0.91%)
dexamethasone 1 MG/ML Oral Solution	154 (0.85%)
dexamethasone 0.1 MG/ML Oral Solution	91 (0.50%)
dexamethasone 0.1 MG/ML Oral Solution [Baycadron]	27 (0.15%)
dexamethasone 0.1 MG/ML Oral Solution [Decadron]	< 20

#### Approach

Given that remdesivir has a well-defined dosage, treatment was able to be defined broadly without relying on any dosage data. Adjustments for dexamethasone treatment in the analysis of outcomes among patients treated with remdesivir were based on binary indicators of whether dexamethasone was administered at any dose. This was considered sufficient for questions focused on the effectiveness of remdesivir. Studies where an understanding of dexamethasone dosage is important may need to take further measures such as performing a sensitivity analysis, as the clinical effects of dexamethasone can vary significantly based on dose, duration and route of administration.

#### Takeaways / suggestions for researchers

Due to inconsistent reporting in drug exposure data, it may be difficult to characterize drug dosing. Depending on the research question being asked, it is possible to be more or less sensitive using the available drug metadata at the expense of selectivity. For example, using the ingredient code and all descendants will be highly sensitive, but will not be specific to an individual dose, strength, or route. If dosing is important, it may be necessary to conduct a study only among data partners that reliably report dosage. When data on dose or route are missing, it is further essential to carefully consider other clinical factors such as bioavailability and/or the extent to which the standard dose has been agreed upon in clinical practice.

#### Duration of drug exposure

#### Goal

Understanding the duration of patient exposure to a particular treatment.

#### Challenge encountered

Within the OMOP data model, a “drug era” is defined as a continuous interval of time over which a patient was exposed to a particular drug. OMOP defines its own derived drug era table based on drug exposures, but relies on certain rules which may not be suitable for all situations. For example, two separate drug exposures separated by a gap of 30 days or less are merged into a single era. This persistence window may be suitable under some circumstances, such as for outpatient exposures to chronic medications, but it is inappropriate for short-term inpatient acute care. Consequently, manually generating drug eras is often required. When doing so, it is important to consider the variability in the drug exposure records found in N3C.

How drug exposure data are presented and some of the issues to consider for remdesivir are illustrated below. Table 4 shows the first common drug exposure scenario encountered, where there are duplicate rows each day representing the same exposure for a particular patient. The exact reason for the duplication can vary; in some cases one row may represent the drug order and the other the administration. This scenario is typically characterized by the presence of a single bounded one-day interval and secondary unbounded interval sharing the same start date. In the scenario below, the total number of exposure days is 5, which is typical for a remdesivir course. Table 5 presents an alternative scenario where drug exposures are combined into contiguous multi-day intervals. It is also the case that some entries may be erroneous, indicating an exposure start date *after* the end date; those entries should be discarded. In the example below, we see that ignoring the unrealistic entry still yields a 5-day course, but that is not always the case.

**Table 4:** Sample single-day drug exposures for a single patient illustrating the presence of duplicate drug exposure start dates

Drug exposure start date	Drug exposure end date
12/31/2020	12/31/2020
12/31/2020	
12/31/2020	
12/31/2020	12/31/2020
12/31/2020	12/31/2020
01/01/2021	
01/01/2021	
01/01/2021	01/01/2021
01/01/2021	01/01/2021
...	...
01/04/2021	01/04/2021

**Table 5:** Sample multi-day drug exposures for a single patient containing reversed start and end dates

Drug exposure start date	Drug exposure end date
2020-11-23	2020-11-24
2020-11-24	2020-11-23
2020-11-24	2020-11-27

The two examples presented are not representative of all possible scenarios encountered in N3C, but they show the two most common documentation paradigms: in some cases exposures were single day with or without end dates, and in other cases drug exposures were in fact presented as multi-day intervals. The main task was dealing with duplicate values, unrealistic start and end dates, other data quality issues, and consolidating contiguous intervals into drug eras.

#### Our approach

**Fig. 3:**
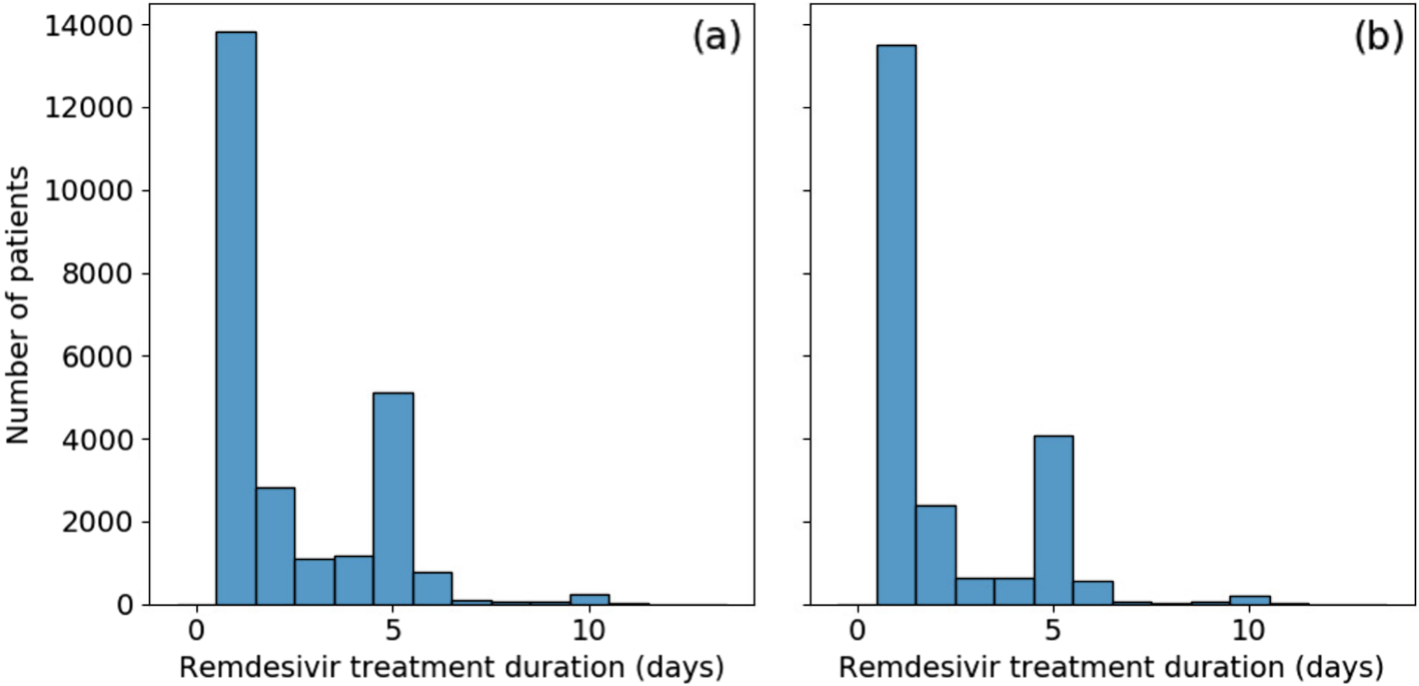
Distribution of remdesivir treatment durations for (**a**) all treated patients and (**b**) patients whose treatment did not terminate with discharge or death

Data were reviewed closely (findings described briefly above), and custom programmatic data cleaning was implemented for every scenario encountered. Remaining open intervals were treated as single day exposures. Consolidating drug exposures did not alleviate all drug era data quality concerns. Figure 3a shows the distribution of remdesivir treatment duration. Over half [55%] of patients appear to have received only a single day of treatment. This is unusual as remdesivir has a very specific recommended course of either 5 or 10 days. There is a secondary large peak at the 5 day mark and a minor additional peak at 10 days. To evaluate the possibility that those treatment courses were terminated early due to mortality or discharge, instances where the final day of treatment coincided with either of those two events were removed. This did not significantly affect the results, as shown in Fig. 3b, with no treatment duration proportion changing by more than 5%.

Further investigation into the drug exposure metadata reveals that over 80% of single-day exposures report “Inferred from claim” for drug type concept name. The metadata for the remaining entries recorded “EHR”, many of which were duplicates, or were empty. These may be the result of incorrectly tagged billing records, but do indicate treatments that were not directly logged into the EHR. The split across data partners was not even; a single data partner was responsible for 73% of the single-day records, with the remaining are split across an additional 10 partners; other data partners did not record single day exposures. Another possibility is that some proportion of these single day exposures are the result of early treatment termination due to adverse drug reactions. However, this was considered unlikely due to the connection with the “inferred from claim” concept name. It is always possible to drop the offending data partners at the expense of statistical power; in this instance, however, the data partner with the largest number of single-day exposures contained 36% of all remdesivir treated patients in our population.

#### Takeaways / suggestions for researchers

The inability to properly resolve treatment durations severely limits the use of methods such as time-dependent Cox regression or marginal structural models for the study of time-varying effects of treatments or exposures. Consultation with clinical experts was also found to be critical to understand realistic use patterns of the medication under study and identify irregular treatment duration reporting. Given the inconsistencies in the data and necessary assumptions to process these data, researchers should consider conducting sensitivity analyses using different assumptions to understand the impact of assumptions on results.

As a final note regarding drug exposures, this discussion has focused on the situation where drug dosing, route of administration, and medication reconciliation were not important. If that information is critical to a particular study, then the drug exposure data require further processing and refinement. This is beyond the scope of this work, but a separate contribution is being prepared where drug exposures are discussed in greater detail.

### Baseline medical history

#### Goal

Assess patient characteristics among remdesivir-treated and non-treated patients and adjust for relevant confounders based on patient histories by summarizing comorbidities identified during a baseline period prior to hospital admission.

#### Challenge encountered

While EHRs can be a rich source of clinical information that is collected prospectively at the time of health care delivery, they often do not contain medical information related to encounters or treatments occurring outside of the contributing healthcare system or prior to an initial encounter. This has been described previously by Lin et al. as EHR-discontinuity [21]. While information exchanges do exist, such as Epic CareEverywhere or Health Information Exchange, there are no guarantees on availability of such records for research purposes. If hospitalized patients typically receive primary health care services from a health system other than that of the admitting hospital, then any prior treatments, medications, immunizations, diagnoses, etc. may not be reflected in the EHR from the system where the COVID hospitalization occurred. Because the patients’ records from other health systems are not included, it may falsely appear that these patients do not have comorbidities or prior treatments. This can result in the misclassification of risk scores such as the Charlson Comorbidity Index [22]. This is distinctly different from continuity issues arising due to lack of care access or patients who avoid seeing medical care. Regardless, comorbidities, prior treatments, or other covariates commonly used for adjustment in observational research are often presumed to be absent if no records are present. This is particularly problematic for studies in acute or critical care settings where admitted patients may have received routine care prior to their admission at an unaffiliated healthcare practice for conditions that may affect outcomes.

**Fig. 4:**
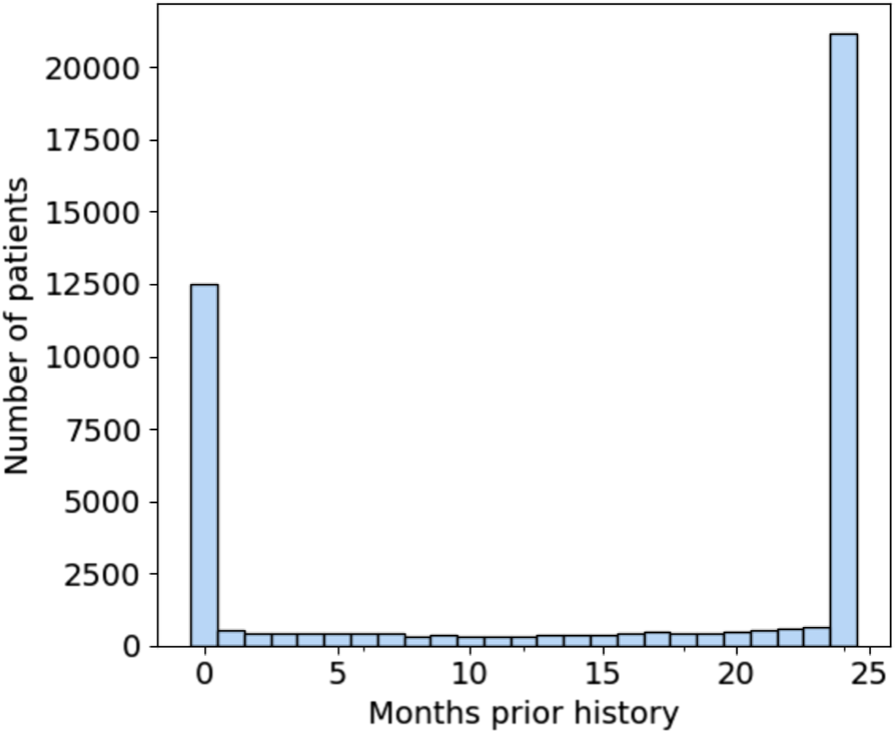
Distribution of months of prior history for first-time COVID 19-related hospitalized patients. Nearly 30% of patients have no recorded visits prior to their admission. Note that N3C patient history lookback was limited to records no older than 1/1/2018. Therefore, all patients with 24 months of history or greater were grouped together

The significance of EHR-discontinuity in N3C was evaluated by first looking at the availability of prior history among the hospitalized patient population. Prior history was defined as the number of months between a patient’s earliest recorded visit of any kind and first COVID-related admission, which would represent the study setting for evaluation of remdesivir effectiveness. Figure 4 shows the resulting distribution of months of prior history in the patient population. There are 12,523 patients, representing nearly 30% of the total population, which have no record of visitation prior to their hospitalization. Of those patients (22.5%) with at least 1 prior visit, the duration of history is approximately uniformly distributed up to 23 months, with a substantial fraction (49%) having ≥24 months (maximum possible in N3C).

The characteristics of patients with no history, 1-23 months of history, and 24 months or greater history are shown in Table 6. There is a statistically significant difference in every characteristic between the three groups. Patients with maximum available history are older (median age, 65 [IQR, 52-77] years vs 60 [IQR, 43-73] and 59 [IQR, 45-71] years of age for 1-23 months and no history, respectively) and are more likely to have documented comorbidities. They are more likely to have recorded medication use for cardiovascular disease (i.e. ACE inhibitors, ARBs, and statins) and are also more likely to be female (11,190 [53%] vs 4894 [50%] and 4862 [39%]). The gender imbalance is consistent with the observation that females are more likely to seek out medical care than males [23].

**Table 6:** Characteristics of COVID-19 related hospitalized patients, by months of prior available history

Variable	No history *N* = 12523^a^	1-23 mo history *N* = 9784^a^	24+ mo history *N* = 21155^a^	***p***-value^b^
**Age**				< 0.001
18-49	3956 (32%)	3154 (32%)	4415 (21%)	
50-59	2485 (20%)	1550 (16%)	3462 (16%)	
60-69	2663 (21%)	2021 (21%)	4595 (22%)	
≥ 70	3419 (27%)	3059 (31%)	8683 (41%)	
**Sex**				< 0.001
Female	4862 (39%)	4894 (50%)	11,190 (53%)	
Male	7661 (61%)	4890 (50%)	9965 (47%)	
**Race**				< 0.001
Asian	566 (4.5%)	359 (3.7%)	603 (2.9%)	
Black or African American	2337 (19%)	2147 (22%)	5106 (24%)	
White	5617 (45%)	4893 (50%)	12,418 (59%)	
Other/Unknown	4003 (32%)	2385 (24%)	3028 (14%)	
**Ethnicity**				< 0.001
Hispanic or Latino	3226 (26%)	2278 (23%)	3107 (15%)	
Not Hispanic or Latino	8133 (65%)	6852 (70%)	17,559 (83%)	
Unknown	1164 (9.3%)	654 (6.7%)	489 (2.3%)	
**BMI**				< 0.001
< 25	2060 (16%)	1944 (20%)	3968 (19%)	
25-29	2756 (22%)	2149 (22%)	4575 (22%)	
30-34	2162 (17%)	1660 (17%)	3462 (16%)	
35-39	1196 (9.6%)	891 (9.1%)	2006 (9.5%)	
≥ 40	1162 (9.3%)	912 (9.3%)	2217 (10%)	
Missing	3187 (25%)	2228 (23%)	4927 (23%)	
**Hypertension**	5605 (45%)	5729 (59%)	15,576 (74%)	< 0.001
**Diabetes**	4173 (33%)	3524 (36%)	9612 (45%)	< 0.001
**CAD**	1380 (11%)	1837 (19%)	6321 (30%)	< 0.001
**CHF**	1690 (13%)	2045 (21%)	6424 (30%)	< 0.001
**COPD**	924 (7.4%)	1168 (12%)	3888 (18%)	< 0.001
**Cerebrovascular disease**	848 (6.8%)	1149 (12%)	4059 (19%)	< 0.001
**CKD**	1637 (13%)	2183 (22%)	7303 (35%)	< 0.001
**Cardiac arrhythmia**	3636 (29%)	3386 (35%)	9942 (47%)	< 0.001
**Tobacco smoking**	770 (6.1%)	1142 (12%)	2506 (12%)	< 0.001
**Malignancy**	744 (5.9%)	1578 (16%)	4452 (21%)	< 0.001
**Prior ACEI treatment**	494 (3.9%)	1431 (15%)	5284 (25%)	< 0.001
**Prior ARB treatment**	391 (3.1%)	1067 (11%)	4071 (19%)	< 0.001
**Prior statin treatment**	829 (6.6%)	2551 (26%)	9475 (45%)	< 0.001
**Ventilated on admission**^c^	2361 (19%)	894 (9.1%)	1852 (8.8%)	< 0.001
**In-hospital mortality**	1785 (14%)	1058 (11%)	2390 (11%)	< 0.001

*Abbreviations*: *CAD* Coronary artery disease, *CHF* Congestive heart failure, *COPD* Chronic obstructive pulmonary disease, *CKD* Chronic kidney disease, *ACEI* Angiotensin-converting enzyme inhibitor, *ARB* Angiotensin 2 receptor blocker

^a^Statistics presented: n (%)

^b^Statistical tests performed: Kruskal-Wallis test; chi-square test of independence

^c^Invasive mechanical ventilation and ECMO were defined as the terms included in the concept sets provided in supplemental Tables S1 and S2

Despite being a significantly younger group and appearing to have lower prevalence of known risk-factors for severe COVID-19 [24, 25], both ventilation on admission (2361 [19%] vs 894 [9.1%] and 1852 [8.8%] for 1-23 and 24+ months respectively) and in-hospital mortality among patients with no history is higher (1785 [14%] vs 1058 [11%] and 2390 [11%] for 1-23 and 24+ months respectively). These patients may represent a heterogeneous mixture of individuals who were previously healthy (truly without comorbidities), patients who received routine care elsewhere (comorbidities present, but data are missing due to healthcare fragmentation), and patients with unmet medical need (comorbidities present but not documented in any health system due to poor access to healthcare). Researchers will need to be aware of potential information bias in estimated treatment effects among hospitalized patients that may be present if differences in EHR continuity are not properly accounted for in analyses.

**Fig. 5:**
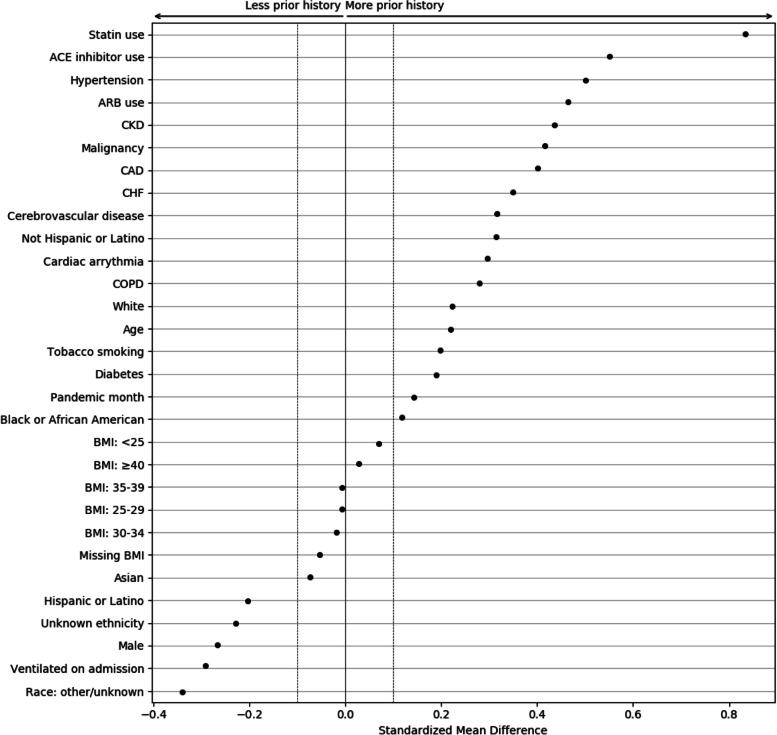
Standardized mean differences between patient characteristics, pandemic timing, and data partner ID, for patients with and without prior history. The largest differences are observed for chronic conditions and their treatments. Missing demographic information such as race and ethnicity also display large differences

**Fig. 6:**
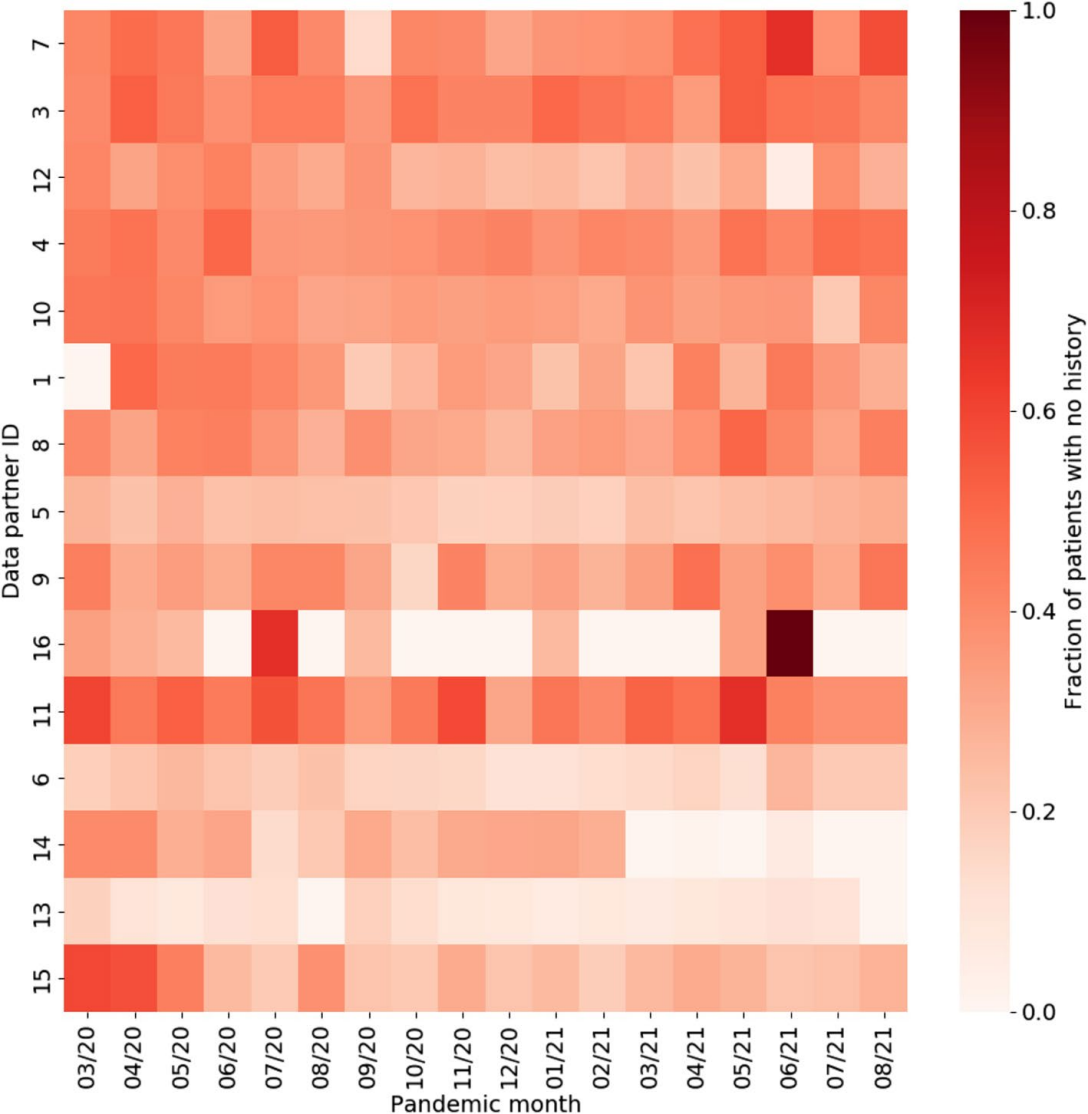
Heatmap showing fraction of patients with no history as a function of pandemic timing and data partner ID

To better understand the magnitude of the differences associated with EHR continuity, the standardized mean differences (SMDs) were calculated between patients with and without any prior history, with magnitude less than 0.1 indicating negligible difference between groups (Fig. 5) [26, 27]. Chronic conditions and related treatments display the greatest differences since they are most likely recorded in a primary care context. We additionally examined potential temporal or location effects. Figure 6 shows the relationship between pandemic timing, data partner ID, and history of missingness in more detail. This is an important step to help identify if there are any specific patterns in prior history missingness that may need to be handled separately. For example, data partners 13 and 6 have a lower level of missingness than others throughout the pandemic while data partner 16 displays a highly irregular pattern of no missingness on most months and a high degree of missingness on others (7/20 and 6/21). These temporal patterns may be the result of upgrades or changes to EHR systems that occurred during the pandemic, the addition or removal of clinics or hospitals in a particular health system, or a potential change in record keeping practices during peak times in the pandemic when healthcare systems were stressed.

#### Approach

Due to EHR discontinuity, it can be challenging to classify traditional baseline health comorbidities in the population of interest. However, in N3C, the availability of laboratory measures from the index COVID-19 hospitalization can be leveraged, relying more heavily on these proximal clinical measures to characterize illness severity and prognosis. When estimating treatment effects, one valuable strategy that does not require an additional modeling is to perform a sensitivity analysis to understand the impact of EHR-continuity on the estimand. The general protocol for performing this analysis would be as follows: first, an initial analysis would be performed including only those patients whose EHR-continuity can be established. Then, the influence of EHR-discontinuity would be assessed by sequentially introducing groups with less prior history; in the N3C, the data include two additional groups, 1-23 months of prior history, and no prior history. As noted previously with the differences in on-admission ventilation and in-hospital mortality, the groups may not reflect the same patient population. If sample size allows, calculating effect estimates separately within groups could also be informative to evaluate the presence of effect measure modification over patient history, or whether treatment effects vary by severity.

#### Takeaways / suggestions for researchers

EHR discontinuity poses a challenge in the use of multi-institution EHR data, limiting the ability to control for confounding by baseline comorbidities. However, severity of illness upon hospitalization is likely one of the most important confounders in studies of COVID-19 treatment. The N3C data do have the unique advantage of having laboratory results and other measurements from the index admission, although, as noted in Table 1, these data may be missing from certain sites. These detailed clinical data measured at index admission can be used as more proximal measures to account for differences in severity of illness across patients, and may be more relevant than many chronic comorbidities that are more difficult to measure [17]. When using the a multi-site EHR database, researchers will need to consider the specific research question at hand, what baseline confounders will be important to measure, and how much history is needed to reliably measure confounders of interest. If proximal variables that are readily available are not sufficient and baseline conditions are necessary to account for, researchers may consider requiring a history (or density) of visits within the database and can refer to prior work that described these strategies in detail [21].

### Clinical outcomes

The effect of remdesivir on in-hospital death, mechanical ventilation, and acute inpatient events were the focus of this work which are all key outcomes of interest in COVID-19 research.

### Mortality

#### Goal

Understanding the risk of mortality among patients hospitalized with COVID-19.

#### Challenge encountered

Patient death data in N3C is primarily obtained from the EHR. Incorporating external mortality data through privacy preserving record linkage (PPRL) has begun. Until this process is complete, only deaths that occur during hospitalizations are guaranteed to be captured. Patient deaths that occur after a patient is discharged home or to another facility are not guaranteed to be captured, resulting in an underestimation of overall mortality. Any outcome involving death can only characterize *in-hospital* mortality. There are rare circumstances where patients’ families report death to a data partner after patient discharge which may be recorded. In those situations, the recorded death date falls outside of the visit date range.

**Fig. 7:**
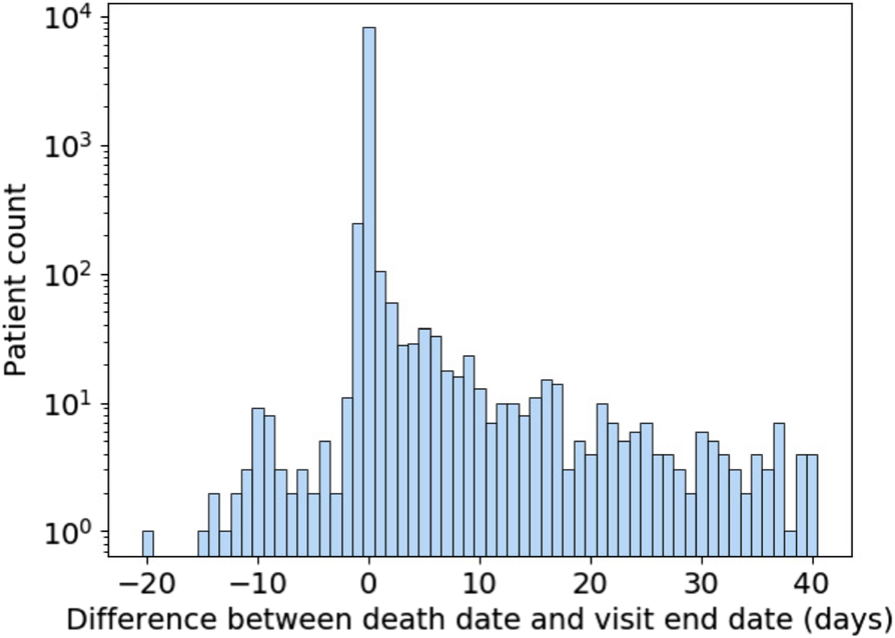
Distribution of difference (in days) between death date and visit end date for patients who died

Data quality is another important consideration when using mortality data in N3C. Figure 7 shows the difference in days between recorded death dates and visit end dates for patients in the selected population who have died. Deaths were excluded for patients who had any record of subsequent visits, which can arise due to billing artifacts. A difference of zero represents deaths that were recorded on the same day as the end of the visit and constitute the majority of deaths. Because timestamps are not always available for visit end and death dates, we limit ourselves to day-level resolution. Depending on the time of day, a small difference in when a death was recorded and when a visit was ended can result in them occurring on different days. As a result, deaths recorded a day before or a day after the visit end date are to be expected. Therefore, it may be justifiable to consider deaths occurring a day after a visit end date as in-hospital deaths.

This leaves both deaths that occur two or more days prior to or after the visit end date. As for the former, they are extremely rare, numbering in the single digits. They can be excluded as part of data quality checking and may be the result of data entry or other sources of error. Deaths which are reported days after a visit end date and are not directly connected to a recorded visit are more numerous. This asymmetry suggests that it is unlikely that the same mechanism is behind both early and late death dates. It may be that some data partners are receiving additional data on deaths which occur outside of their facilities following hospitalization. However, since there are no specific guidelines or requirements for out-of-hospital mortality reporting within N3C, it cannot be relied on for analysis. Therefore, it is best to treat those patients as not having experienced in-hospital mortality for that visit because this supplementary death data was not systematically available.

#### Approach

Due to the inability to reliably study deaths that occur out-of-hospital, the outcome was carefully defined for this analysis as inpatient death within 28 days. Patients who died more than a day before discharge were dropped, and mortality for patients who died greater than 1 day past their visit end date was not considered.

#### Takeaways/suggestions for researchers

Researchers should be familiar with the limitations in studying mortality in multi-site EHR repositories. Unless complete linkage to external mortality data is present, the data only reliably capture deaths that occur during hospitalization. Any studies reporting risks of mortality using these data will need to be explicit in how mortality is defined, and consider the implications of studying only in-hospital mortality. To assess the impact of this limitation on estimates of risk, researchers can consider conducting sensitivity analyses, comparing mortality risk estimates when patients are censored at a fixed follow-up duration. Alternatively, discharge can be directly treated as a competing risk using competing risk methods such as the Fine-Gray subdistribution hazard model or cumulative incidence functions [28–30]. Within N3C, post-discharge mortality data has recently become available through PPRL which largely addresses this issue. However, it still remains a common problem in EHR-based research where external linkage may not be available.

### Acute inpatient events

#### Goal

Understanding how to distinguish acute events related to complications of initial cause of hospitalization from adverse events of treatment.

#### Challenge encountered

Common acute events occurring during hospitalization range from DVT/PE, myocardial infarction (MI), stroke, pulmonary edema, and allergic reactions to common infections such as bacterial pneumonia, urinary tract infection (UTI), or sepsis. Identifying such acute events accurately from EHR data can be challenging for a few reasons. First, ensuring that these events have acute onset and do not represent previous medical history carried forward into the visit requires detailed record checking. While OMOP does provide a field for supplying condition status (eg. “Primary diagnosis”, “Secondary diagnosis”, “Final diagnosis (discharge)”, “Active”, “Resolved”, etc.…), it is rarely populated in N3C and thus not suitable for analysis. Second, parsing out diagnoses occurring as a response to treatment versus those that are the reason for receiving a treatment is complicated by the absence of sufficiently high temporal resolution and detailed clinical information which is typically buried in unstructured EHR data. Third, suspected or differential diagnoses can potentially be misclassified when identifying an acute event by diagnosis codes.

#### Approach

First, the presence of the outcome prior to hospitalization is checked. Due to variability in EHR entry, some data partners record previous medical history daily during a patient’s visit, which eliminates the ability to differentiate between a new or recurring event. For example, a patient with a previous medical history of MI may have this diagnosis in their medical record. For each day during the patient’s hospitalization, their medical history is carried forward and displays a diagnosis of MI. However, this does not represent the patient having a new MI on a daily basis. Such scenarios can be identified by calculating the number of events recorded per day throughout the visit. If the event of interest occurs at least daily, these patients cannot be considered to have experienced that acute event.

Timing of the outcome is also important. When assessing the safety and effectiveness of a treatment, confirming that the acute event occurred as a response to treatment is required. Although identifying cause and effect is challenging, steps can be taken to mitigate errors. Once patients who were identified to have had a previous medical history of an event were excluded, it was ensured that the event occurred after the treatment of interest. More generally, appropriate time windows should be considered based on anticipated effects of the drug or procedure. Any patients who have an outcome prior to treatment should be excluded.

#### Takeaways/suggestions for researchers

Researchers will require detailed assessment of acute event outcomes to ensure they do not represent prior events carried forward. Additional data can be helpful to ensure accurate detection of acute events. Confirming events with lab results when available can further increase confidence in detecting the outcome. For example, MI diagnosis with corresponding elevated troponin can improve specificity of capture. Events without explicit lab findings can be supported with related procedures. The level of sensitivity vs specificity is dependent on the study objectives and can be further assessed through a sensitivity analysis.

### Composite outcomes

**Fig. 8:**
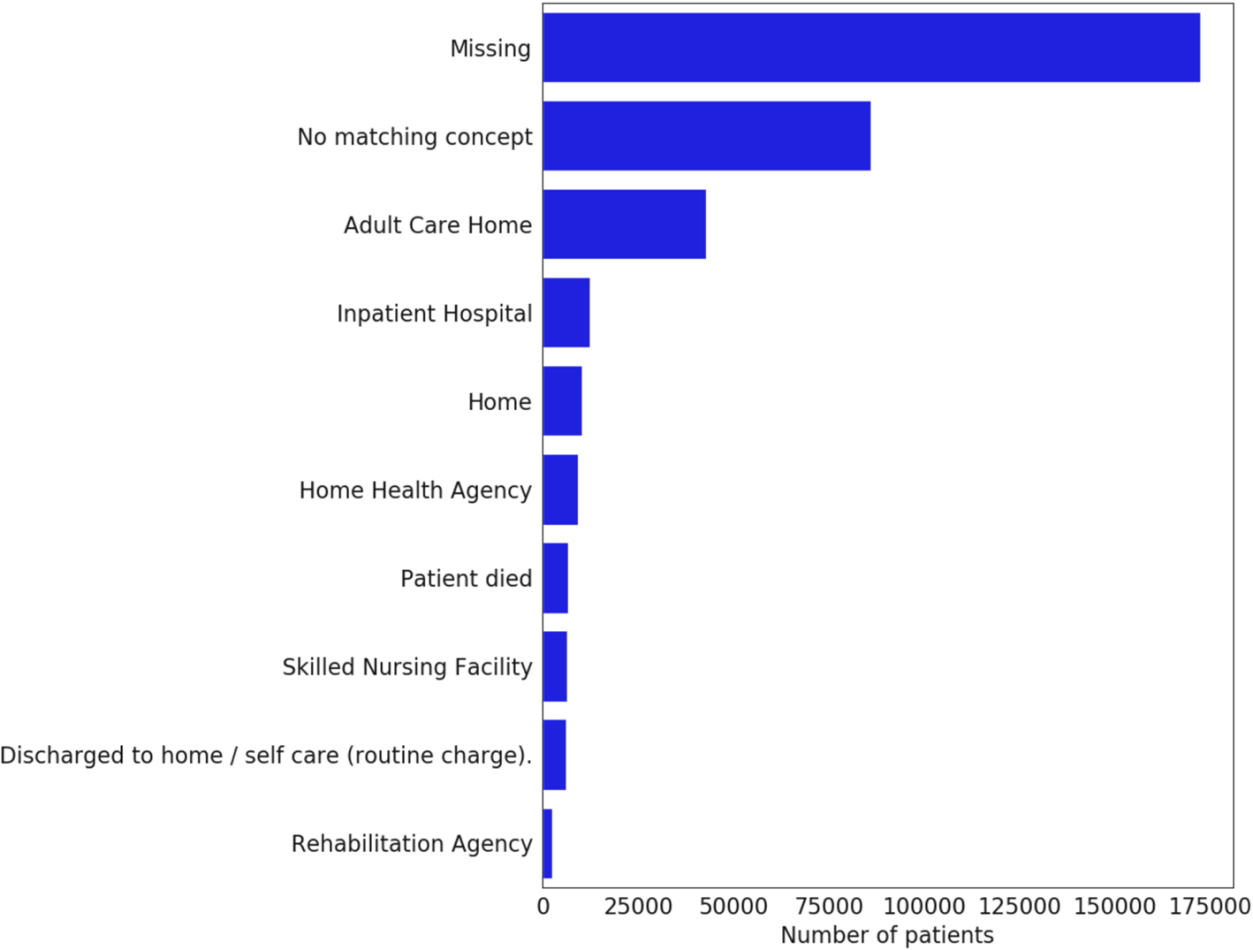
Top ten most frequently occurring discharge dispositions across all data partners

Composite outcomes, such as in-hospital death or discharge to hospice, incorporate information from the discharge disposition. Unfortunately, discharge disposition reporting is very rare, with some CDMs lacking support altogether (Fig. 8). Therefore, they are best avoided, or, if strictly necessary, should be limited to data partners who undergo consistency and completion checks.

## Discussion

N3C offers a significant step forward in providing access to integrated national-level EHR data which serves as a source for RWE that can help guide both public health policy, future prospective controlled clinical trials, and clinical decision making. It is part of a broader trend in which sources of RWD are consolidated into larger repositories for research purposes. This enables large-scale validation of findings across multiple health systems, the ability to achieve sufficient statistical power when investigating treatments and conditions with low prevalence, and improve equitability and representation of underserved hospitals and patient demographics in treatment effect studies. In addition, large data sets provide an opportunity for the deployment of machine learning methods while mitigating the risk of overfitting. As a centralized repository, N3C aggregates and harmonizes data in a single location. While this comes with its own challenges, it allows the data to be queried at the row level and enables detailed centralized investigations into data quality such as those presented here. This is in contrast to federated data models which allow data to be queried in aggregate, but rely on data curation at the local level [7]. Nevertheless, operationalizing definitions for key clinical events can be complicated by limitations in site reporting and a loss of granularity through the use of a common data model to harmonize data across partners. The advantages of harmonization, however, outweighs the loss of granularity when addressing many research questions. The efficiency of centralization is also a significant advantage and was witnessed through the course of this study. For example, a number of sites using the ACT CDM initially did not have the ability to report vitals, but subsequently these data were made available due to continuous feedback during the review process. Sites are provided with data quality metrics and furnished with support to help address gaps or deficiencies, when possible.

The promise of data enclaves such as N3C are clear, yet it remains imperative to consider the limitations of the data and ensure that they are fit for purpose. Data missingness, for example, remains a significant analytical challenge. It may be reasonable to suspect that many of the lab measurements investigated in this study are informatively missing, in that they are clinically indicated (and thus ordered) only in the most severe patient cases. Yet, this may be confounded by individual data-contributing health systems’ respective capacities to obtain these measures at a given point in time (e.g., clinicians faced with an overwhelming caseload during pandemic ‘peaks’ may have inferred a level of clinical severity in some patients without ordering/recording such measures once seeing it repeatedly in other clinically similar cases). This suspected mixture of missing at random (MAR) and missing not-at-random (MNAR) mechanisms, likely varying over time within each data partner health system, presents a challenge to data analysts who ⏤ in trying to delineate between instances of informatively vs. inadvertently missing values ⏤ cannot directly access subject-matter experts (such as clinical care providers experienced in each health system during each time interval under study) to form the reasonably defensible assumptions required, assumptions not verifiable from EHR data alone [31]. With many severity-of-illness indicators, it is common to create derived factors and missing data indicators. For example, body temperature may be used to create a three-level factor: “Normal temperature (<= 38 deg C)”, “Fever (> 38 deg C)”, and “Missing.” However, this approach has been shown to exhibit severe bias even with MAR data [32]. The danger of this approach becomes even more evident when glancing at data missingness as a function of data partner and time shown previously in Fig. 2. A more principled variation on this approach is forming distinct (collections of) additional variable(s) indicating the missing value status for each variable in any given analysis; conditioning on these indicators in downstream analyses would make explicit the assumptions inherent to how those with missing values may be plausibly assumed to differ from those without missing values for the variables prone to missingness (and would inform analysts how to elicit plausible assumptions from domain experts, by considering these ‘full’ data).

Excluding sites which are major contributors to data missingness does not eliminate all missing data, and there are outstanding issues that still need to be addressed. Here, considering the different possible mechanisms responsible for missingness is necessary. One contribution to overall missingness involves mechanisms reasonably assumed to be MCAR (an effectively random sample of individuals from included sites still lack values expected to have been recorded), while another likely contribution is disease severity. This mechanism is closely related to what is termed in the statistical literature as outcome-dependent observation processes [33–35]. Labs are usually ordered in sets, with complete blood count (CBC) and basic metabolic panel (BMP) being the most common, followed by complete metabolic panel (CMP). For COVID-19 positive patients, some providers may add CRP, d-dimer, and ferritin to CBC and BMP panels. Importantly, all of these orders vary depending on patient condition, provider practice, and standards of care - all of which may vary throughout the course of the pandemic; thus more tenable assumptions can be adopted (at least approximately, to mitigate bias) after considering how analyses incorporate care settings (from healthcare-system down to clinic/provide levels) as well as calendar time as proxy measures for such systematic differences. Similar MNAR mechanisms have been identified in end-of-life care studies where questionnaire missingness is related to poorer health status [36].

In isolation, the labs as described above are MNAR since the decisions made to order specific labs are often based on existing clinical information. If the probability of observing a covariate can be assumed to not depend on its value after conditioning on other observables, then that covariate is considered MAR. This is often difficult to assume – let alone empirically verify from available data – in practice, without supplemental auxiliary data, but a detailed look at key conditional distributions combined with domain expertise can justify the adoption of MAR assumptions. In that case, a number of techniques including multiple imputation and inverse probability weighting exist to handle said missingness under specific assumptions, though special attention needs to be paid to both the model specification and algorithm [37]. Finally, although complete-case analysis is used by some practitioners in these settings, omitting records with any missing data among variables associated with exposures/confounders or outcomes is known to bias effects estimates and, at best, reduce precision [32, 38].

Complete drug exposures along with associated details such as dose and route of administration are not always available, constraining the possible study designs or requiring a tradeoff between sensitivity and specificity in defining treatment. In our analysis, for example, we were only concerned with adjusting for dexamethasone treatment as it is commonly administered and has been shown as part of the RECOVERY trial to lower mortality in hospitalized patients receiving respiratory support [39]. However, a dedicated investigation into the effectiveness of dexamethasone may involve more subsetting of data based on availability of dosage and route of administration. Alternative explanations for observed drug exposure patterns should also receive consideration. The witnessed distribution of drug eras for remdesivir in our study may be consistent with some artifactual ‘coarsening’ mechanism at play in how actual drug exposures in each patient case may, depending on coding and CDM-mapping practice that vary by data partner, tend toward inclusion of ‘rounder’ numbers such as 1, 5, and 10 days [40]. Additionally worth reiterating is the possibility that treatment with remdesivir was terminated early, and therefore had unexpected durations, due to drug reactions and side effects that outweighed benefits of treatment.

Looking more broadly, being limited to EHR data means no enrollment information demarcating a specific time period during which records are known to be complete is available; there are no guarantees on patient-level completeness. Therefore, estimating EHR continuity, as we have outlined above, is a crucial part of mitigating the resulting bias. It cannot be assumed that the lack of a given baseline comorbidity or therapy is evidence of its absence, particularly for chronic conditions and medications, unless EHR continuity can be established. This is also true for COVID-19 vaccination, which is an important exposure for COVID-19-related studies. There is no explicit indicator of non-vaccination, and the widespread availability of vaccines can further contribute to data fragmentation. It does appear however that some institutions may synchronize vaccination records with their state’s vaccine registry, which provides one strategy for assessing the completeness of an institution’s vaccine records [41]. Given the significant potential for information bias due to EHR-discontinuity, some have proposed using predictive modeling to identify patients with high EHR-continuity [21]. Furthermore, carefully designed methodological frameworks are needed to handle selection bias (the sickest patients often have the most complete records) which can arise when enforcing data completeness for the EHR data [42]. There are ongoing efforts within N3C to link to CMS claims which will address many of these concerns for at least a specific patient population. Additionally, data mining unstructured data such as clinical notes may provide more comprehensive information on baseline comorbidities, particularly for patients which lack a prior history with the admitting health system.

Suitable outcomes for evaluation are generally limited by the availability of data in EHRs and more specifically limited by both the OMOP data model and data partner reporting. ICU admissions, for example, cannot be resolved from the visit-level information available, notwithstanding the possibility that some sites repurposed non-ICUs to serve as ICUs during surges in COVID-19 patients. Additionally, despite the popularity of composite outcomes, such as death or discharge to hospice, we find that most data partners do not provide discharge disposition. Perhaps most importantly with regards to outcomes is the lack of availability of mortality data outside the EHR, for the time being, which underestimates overall mortality and restricts investigations to in-hospital mortality alone. Although the use of survival methods may not be the most appropriate choice for patients who are hospitalized with critical illness [43], they are still nonetheless quite widely used. For survival analysis, being limited to in-hospital mortality alone has important implications. Patients who are discharged from the hospital are typically discharged due to recovery, or to a different care facility due to disease severity. In either case, the risk of death among discharged patients is not the same as those patients who remain hospitalized. Censoring patients at discharge introduces a differential risk of death between censored and non-censored observations, violating the non-informative censoring assumption necessary for common survival models such as Cox proportional hazards. This violation can be addressed by censoring all patients after a fixed time period, known as the “best-case” or “best-outcome” approach, which assumes discharged patients have survived until the end of the observation period [30]. Alternatively, one can treat discharge as a competing outcome and rely on the subdistribution hazard function or other methods for competing-risk analysis [32]. The most direct remedy would be to link patient records through PPRL to ancillary sources of mortality data to capture deaths post-discharge. This may be particularly important in view of published data suggesting an increased risk of mortality for many months following hospitalization for COVID-19 [44]. A summary of the issues presented is shown in Table 7.

**Table 7:** Summary of challenges presented along with possible solutions

Challenge	Possible solution(s)
Source-specific variability in data availability	• Cluster data sources based on relevant study variables and eliminate those with insufficient data. • Investigate possible temporal missingness patterns and evidence of MNAR data. • Potentially leverage relevant techniques such as multiple imputation and inverse probability weighting to handle remaining missing data.
Unreconciled drug exposure intervals	• Aggregate contiguous drug exposure intervals into single drug eras. • Residual open-ended intervals may not allow for time-varying analysis and may only be suitable for analysis as point exposures.
Absence of baseline medical history	• Perform a sensitivity analysis to understand the impact of EHR-continuity on the estimand. • Consider incorporating prognostic factors proximal to the outcome into the model.
Limited availability of out-of-hospital mortality data	• Consider a sensitivity analysis on censoring time for discharged patients. • Employ competing risk analysis analysis with discharge and in-hospital mortality as competing risks.
Previous medical history carried forward in EHR data	• Calculate the number of events recorded per day throughout the visit for an outcome of interest. • Determine if treatment preceded the outcome or if it is an artifact.

These pervasive issues have been noted across a number of multi-site EHR repositories [45, 46]. In Optum De-identified COVID-19 EHR, Chawla et al. notes that missing data is MNAR due to the urgency of the pandemic and can affect measured outcomes [47]. Dependence on diagnostic and procedural codes may result in underreporting of events, and mortality rates can also be underestimated. Another analysis using the COVID-19 Research Database explains that only associations rather than causality can be determined using available medical record data as unmeasured confounders can mask true links between outcomes [48].

With the proper strategies and data quality considerations, N3C is particularly well suited to investigate treatment effectiveness in hospitalized patients. It contains rich and detailed clinical data such as laboratory results, vital signs, and other measurements. These observations can serve as proximal measures to account for differences in severity of illness across patients, enable patient phenotyping and confounding adjustment, and may be more important and relevant than many chronic comorbidities that may be more difficult to measure. Additionally, data in N3C are routinely updated with little to no time lag, which is critical when pandemic conditions are changing rapidly and new variants of the SARS-CoV-2 virus are emerging. With the appropriate treatment of data quality issues as outlined in this paper, in addition to robust study design, N3C has demonstrated its central importance in serving as a source of RWE for COVID-19.

## Conclusions

The creation of the N3C Data Enclave and the centralization of EHRs from data partners across the country enables a wide range of RWE research aimed at better understanding treatments and health impacts of COVID-19. As with all observational research, it is important that researchers are judicious in understanding the data they are analyzing and defining research questions that are both clinically important and feasible to address using the available data.

We share lessons learned in the context of using N3C to evaluate remdesivir use in hospitalized COVID-19 patients. We found it was necessary to spend considerable time evaluating and curating the data, and selecting data partners with sufficient data quality in the elements required to address the specific question at hand. Given EHR-related data limitations, we needed to make assumptions and design decisions when operationalizing concepts of interest. We emphasize the importance of transparency in observational research and urge researchers to be explicit in how concepts are defined, and how various necessary assumptions may introduce bias. Lastly, we encourage researchers to conduct sensitivity analyses to assess the robustness of the results to assumptions made in the study design process [49].

The N3C Data Enclave is a major contribution to public health making possible a breadth of research addressing the COVID-19 pandemic. To maintain public trust in scientific research, we must be thoughtful in using this resource responsibly by asking questions falling within the scope of the data, and form conclusions that are supported by the analysis.

## Supplementary Information

**Additional file 1: Table S1.** List of terms used to create ECMO concept set. **Table S2.** List of terms used to create invasive mechanical ventilation concept set.

## Data Availability

The analyses described in this publication were conducted with data or tools accessed through the NCATS N3C Data Enclave covid.cd2h.org/enclave and supported by CD2H - The National COVID Cohort Collaborative (N3C) IDeA CTR Collaboration 3U24TR002306-04S2 NCATS U24 TR002306. This research was possible because of the patients whose information is included within the data from participating organizations (covid.cd2h.org/dtas) and the organizations and scientists (covid.cd2h.org/duas) who have contributed to the on-going development of this community resource. Enclave data is protected, and can be accessed for COVID-related research with an approved (1) IRB protocol and (2) Data Use Request (DUR). Enclave and data access instructions can be found at https://covid.cd2h.org/for-researchers; all code used to produce the analyses in this manuscript is available within the N3C Enclave to users with valid login credentials to support reproducibility.

## Abbreviations

EHR: Electronic health records
RWD: Real world data
RWE: Real world evidence
N3C: National COVID Cohort Collaborative
NCATS: National Center for Advancing Translational Sciences
CDM: Common data model
PaO2: Partial arterial oxygen pressure
FiO2: Fraction of inspired oxygen
WBC: White blood count
CRP: C-reactive protein
IL-6: Interleukin-6
SpO2: Oxygen saturation
ETL: Extract/transform/load
LDS: Limited data set
MNAR: Missing not at random
MAR: Missing at random
MCAR: Missing completely at random
CAD: Coronary artery disease
CHF: Congestive heart failure
COPD: Chronic obstructive pulmonary disease
CKD: Chronic kidney disease
ACEI: Angiotensin-converting enzyme inhibitor
ARB: Angiotensin 2 receptor blocker
SMD: Standardized mean differences
PPRL: Privacy preserving record linkage
DVT: Deep vein thrombosis
PE: Pulmonary embolism
MI: Myocardial infarction
UTI: Urinary tract infection
CBC: Complete blood count
BMP: Basic metabolic panel
CMP: Complete metabolic panel
