# Supplementary material for: Data quality considerations for evaluating COVID-19 treatments using real world data: learnings from the National COVID Cohort Collaborative (N3C)
Source: BMC Med Res Methodol. 2023 Feb 17;23:46. doi: 10.1186/s12874-023-01839-2 (PMC9936475; doi:10.1186/s12874-023-01839-2)
Supplement: Supplementary file 1 — Additional file 1: Table S1. List of terms used to create ECMO concept set. Table S2. List of terms used to create invasive mechanical ventilation concept set. [file 12874_2023_1839_MOESM1_ESM.docx]

# Supplementary Information

**Table S1.** List of terms used to create ECMO concept set.

| 46257680 | Extracorporeal membrane oxygenation (ECMO)/extracorporeal life support (ECLS) provided by physician; removal of central cannula(e) by sternotomy or thoracotomy, 6 years and older |
| --- | --- |
| 46257513 | Extracorporeal membrane oxygenation (ECMO)/extracorporeal life support (ECLS) provided by physician; reposition peripheral (arterial and/or venous) cannula(e), percutaneous, birth through 5 years of age (includes fluoroscopic guidance, when performed) |
| 46257439 | Extracorporeal membrane oxygenation (ECMO)/extracorporeal life support (ECLS) provided by physician; reposition of central cannula(e) by sternotomy or thoracotomy, birth through 5 years of age (includes fluoroscopic guidance, when performed) |
| 2002247 | Extracorporeal membrane oxygenation [ECMO] |
| 46257510 | Extracorporeal membrane oxygenation (ECMO)/extracorporeal life support (ECLS) provided by physician; daily management, each day, veno-venous |
| 46257685 | Extracorporeal membrane oxygenation (ECMO)/extracorporeal life support (ECLS) provided by physician; removal of central cannula(e) by sternotomy or thoracotomy, birth through 5 years of age |
| 46257400 | Insertion of left heart vent by thoracic incision (eg, sternotomy, thoracotomy) for ECMO/ECLS |
| 37206603 | Venovenous extracorporeal membrane oxygenation |
| 46257729 | Extracorporeal membrane oxygenation (ECMO)/extracorporeal life support (ECLS) provided by physician; reposition peripheral (arterial and/or venous) cannula(e), open, birth through 5 years of age (includes fluoroscopic guidance, when performed) |
| 46257467 | Extracorporeal membrane oxygenation (ECMO)/extracorporeal life support (ECLS) provided by physician; reposition peripheral (arterial and/or venous) cannula(e), percutaneous, 6 years and older (includes fluoroscopic guidance, when performed) |
| 46257438 | Extracorporeal membrane oxygenation (ECMO)/extracorporeal life support (ECLS) provided by physician; insertion of peripheral (arterial and/or venous) cannula(e), open, 6 years and older |
| 1531630 | Extracorporeal Oxygenation, Membrane, Peripheral Veno-venous |
| 46257466 | Extracorporeal membrane oxygenation (ECMO)/extracorporeal life support (ECLS) provided by physician; insertion of peripheral (arterial and/or venous) cannula(e), percutaneous, 6 years and older (includes fluoroscopic guidance, when performed) |
| 46257682 | Extracorporeal membrane oxygenation (ECMO)/extracorporeal life support (ECLS) provided by physician; initiation, veno-arterial |
| 1531632 | Extracorporeal Oxygenation, Membrane, Central |
| 46257399 | Extracorporeal membrane oxygenation (ECMO)/extracorporeal life support (ECLS) provided by physician; removal of peripheral (arterial and/or venous) cannula(e), open, 6 years and older |
| 46257440 | Extracorporeal membrane oxygenation (ECMO)/extracorporeal life support (ECLS) provided by physician; reposition central cannula(e) by sternotomy or thoracotomy, 6 years and older (includes fluoroscopic guidance, when performed) |
| 46257398 | Extracorporeal membrane oxygenation (ECMO)/extracorporeal life support (ECLS) provided by physician; insertion of central cannula(e) by sternotomy or thoracotomy, 6 years and older |
| 46257684 | Extracorporeal membrane oxygenation (ECMO)/extracorporeal life support (ECLS) provided by physician; reposition peripheral (arterial and/or venous) cannula(e), open, 6 years and older (includes fluoroscopic guidance, when performed) |
| 46257441 | Arterial exposure with creation of graft conduit (eg, chimney graft) to facilitate arterial perfusion for ECMO/ECLS (List separately in addition to code for primary procedure) |
| 46257511 | Extracorporeal membrane oxygenation (ECMO)/extracorporeal life support (ECLS) provided by physician; daily management, each day, veno-arterial |
| 44811012 | Fluoroscopy guided percutaneous insertion of cannula for extracorporeal membrane oxygenation |
| 37206602 | Arteriovenous extracorporeal membrane oxygenation |
| 46257586 | Extracorporeal Membrane Oxygenation or Extracorporeal Life Support Services and Procedures |
| 2787820 | Extracorporeal Supersaturated Oxygenation, Intermittent |
| 2787821 | Extracorporeal Hyperbaric Oxygenation, Continuous |
| 46257544 | Extracorporeal membrane oxygenation (ECMO)/extracorporeal life support (ECLS) provided by physician; removal of peripheral (arterial and/or venous) cannula(e), open, birth through 5 years of age |
| 46257397 | Extracorporeal membrane oxygenation (ECMO)/extracorporeal life support (ECLS) provided by physician; insertion of central cannula(e) by sternotomy or thoracotomy, birth through 5 years of age |
| 4052536 | Extracorporeal membrane oxygenation |
| 46257468 | Extracorporeal membrane oxygenation (ECMO)/extracorporeal life support (ECLS) provided by physician; removal of peripheral (arterial and/or venous) cannula(e), percutaneous, 6 years and older |
| 46257469 | Removal of left heart vent by thoracic incision (eg, sternotomy, thoracotomy) for ECMO/ECLS |
| 46257512 | Extracorporeal membrane oxygenation (ECMO)/extracorporeal life support (ECLS) provided by physician; insertion of peripheral (arterial and/or venous) cannula(e), percutaneous, birth through 5 years of age (includes fluoroscopic guidance, when performed) |
| 46257683 | Extracorporeal membrane oxygenation (ECMO)/extracorporeal life support (ECLS) provided by physician; insertion of peripheral (arterial and/or venous) cannula(e), open, birth through 5 years of age |
| 44515635 | Extracorporeal membrane oxygenation |
| 46257730 | Extracorporeal membrane oxygenation (ECMO)/extracorporeal life support (ECLS) provided by physician; removal of peripheral (arterial and/or venous) cannula(e), percutaneous, birth through 5 years of age |
| 4338595 | Cardiac support using extracorporeal membrane oxygenation circuitry |
| 1531631 | Extracorporeal Oxygenation, Membrane, Peripheral Veno-arterial |
| 46257543 | Extracorporeal membrane oxygenation (ECMO)/extracorporeal life support (ECLS) provided by physician; initiation, veno-venous |
| 46257585 | Extracorporeal membrane oxygenation (ECMO)/extracorporeal life support (ECLS) provided by physician |
| 37206601 | Venoarterial extracorporeal membrane oxygenation |

**Table S2.** List of terms used to create invasive mechanical ventilation concept set.

| 4229714 | Mechanical ventilation weaning response |
| --- | --- |
| 4026054 | Changing endotracheal tube |
| 2805870 | Extracorporeal or Systemic Assistance and Performance @ Physiological Systems @ Assistance @ Respiratory @ Less than 24 Consecutive Hours @ Ventilation |
| 4179373 | Insertion of endotracheal tube using laryngoscope |
| 2893766 | Extracorporeal or Systemic Assistance and Performance @ Physiological Systems @ Performance @ Respiratory @ Less than 24 Consecutive Hours @ Ventilation |
| 44808555 | Provision of mechanical ventilator |
| 4301549 | Ventilator care and adjustment |
| 4058031 | Endotracheal intubation, emergency procedure |
| 2867784 | Extracorporeal or Systemic Assistance and Performance @ Physiological Systems @ Assistance @ Respiratory @ 24-96 Consecutive Hours @ Ventilation |
| 2788023 | Assistance with Respiratory Ventilation, 24-96 Consecutive Hours |
| 2314000 | Ventilation assist and management, initiation of pressure or volume preset ventilators for assisted or controlled breathing; hospital inpatient/observation, initial day |
| 42738852 | Ventilation assist and management, initiation of pressure or volume preset ventilators for assisted or controlled breathing; first day (Deprecated) |
| 44791135 | Ventilatory support |
| 4335481 | Orotracheal intubation |
| 2800859 | Extracorporeal or Systemic Assistance and Performance @ Physiological Systems @ Performance @ Respiratory @ 24-96 Consecutive Hours @ Ventilation |
| 4031379 | Artificial ventilation finding |
| 40481547 | Dependence on ventilator |
| 37116698 | Insertion of double lumen tracheobronchial tube |
| 4237618 | Ventilator care |
| 4168966 | Endotracheal tube present |
| 2108681 | Patient receiving care in the intensive care unit (ICU) and receiving mechanical ventilation, 24 hours or less (CRIT) |
| 2788038 | Respiratory Ventilation, Greater than 96 Consecutive Hours |
| 2788028 | Assistance with Respiratory Ventilation, Greater than 96 Consecutive Hours |
| 46273390 | Dependence on respirator |
| 4337615 | Orotracheal fiberoptic intubation |
| 2834015 | Extracorporeal or Systemic Assistance and Performance @ Physiological Systems @ Performance @ Respiratory @ Greater than 96 Consecutive Hours @ Ventilation |
| 44509482 | Other specified ventilation support |
| 4235361 | Hyperventilation therapy for traumatic brain injury |
| 4287922 | Weaning from mechanically assisted ventilation commenced |
| 4056812 | Laryngeal intubation for inhalation |
| 4119642 | Awake intubation |
| 4337616 | Nasotracheal intubation |
| 4335585 | Endobronchial intubation |
| 4082243 | Emergency laryngeal intubation |
| 44515633 | Other specified intubation of trachea |
| 4013354 | Insertion of endotracheal tube |
| 4337617 | Nasotracheal fiberoptic intubation |
| 4251737 | Ventilator care management |
| 4287921 | Retrograde intubation |
| 4140765 | Digital assisted intubation |
| 765576 | Orotracheal intubation using bougie device |
| 2314001 | Ventilation assist and management, initiation of pressure or volume preset ventilators for assisted or controlled breathing; hospital inpatient/observation, each subsequent day |
| 4308797 | Trial for spontaneous breathing |
| 4232891 | Mechanical ventilation response |
| 4325601 | Complication of ventilation therapy |
| 4339623 | Oral intubation awake |
| 4303945 | Tracheal intubation through a laryngeal mask airway |
| 2788037 | Respiratory Ventilation, 24-96 Consecutive Hours |
| 42738853 | Ventilation assist and management, initiation of pressure or volume preset ventilators for assisted or controlled breathing; subsequent days (Deprecated) |
| 4353715 | Ventilator finding |
| 4134538 | Unintended endobronchial intubation |
| 45889042 | Ventilator Management |
| 2788036 | Respiratory Ventilation, Less than 24 Consecutive Hours |
| 2007912 | Other intubation of respiratory tract |
| 4149878 | Transglottic catheterization of trachea |
| 4335584 | Nasal intubation awake |
| 2106469 | Intubation, endotracheal, emergency procedure |
| 4283807 | Intubation of larynx |
| 4230167 | Artificial respiration |
| 4174085 | Dual pressure spontaneous ventilation support weaning protocol |
| 4335583 | Tracheal intubation using rigid bronchoscope |
| 4080957 | Endotracheal respiratory assistance |
| 45887795 | Ventilation assist and management, initiation of pressure or volume preset ventilators for assisted or controlled breathing |
| 2788018 | Assistance with Respiratory Ventilation, Less than 24 Consecutive Hours |
| 4219858 | Problem with patient ventilator |
| 37116689 | Insertion of endotracheal ventilation catheter |
| 4072633 | Weaning from mechanically assisted ventilation |
| 40487536 | Intubation of respiratory tract |
| 4134853 | Weaning from mechanically assisted ventilation continued |
| 4259233 | Ventilator care assessment |
| 4337045 | Blind nasal intubation |
| 2813710 | Extracorporeal or Systemic Assistance and Performance @ Physiological Systems @ Assistance @ Respiratory @ Greater than 96 Consecutive Hours @ Ventilation |
